# Supplementary figures and images for: Systems Analysis Reveals Ageing-Related Perturbations in Retinoids and Sex Hormones in Alzheimer’s and Parkinson’s Diseases
Source: Biomedicines. 2021 Sep 24;9(10):1310. doi: 10.3390/biomedicines9101310 (PMC8533098; doi:10.3390/biomedicines9101310)

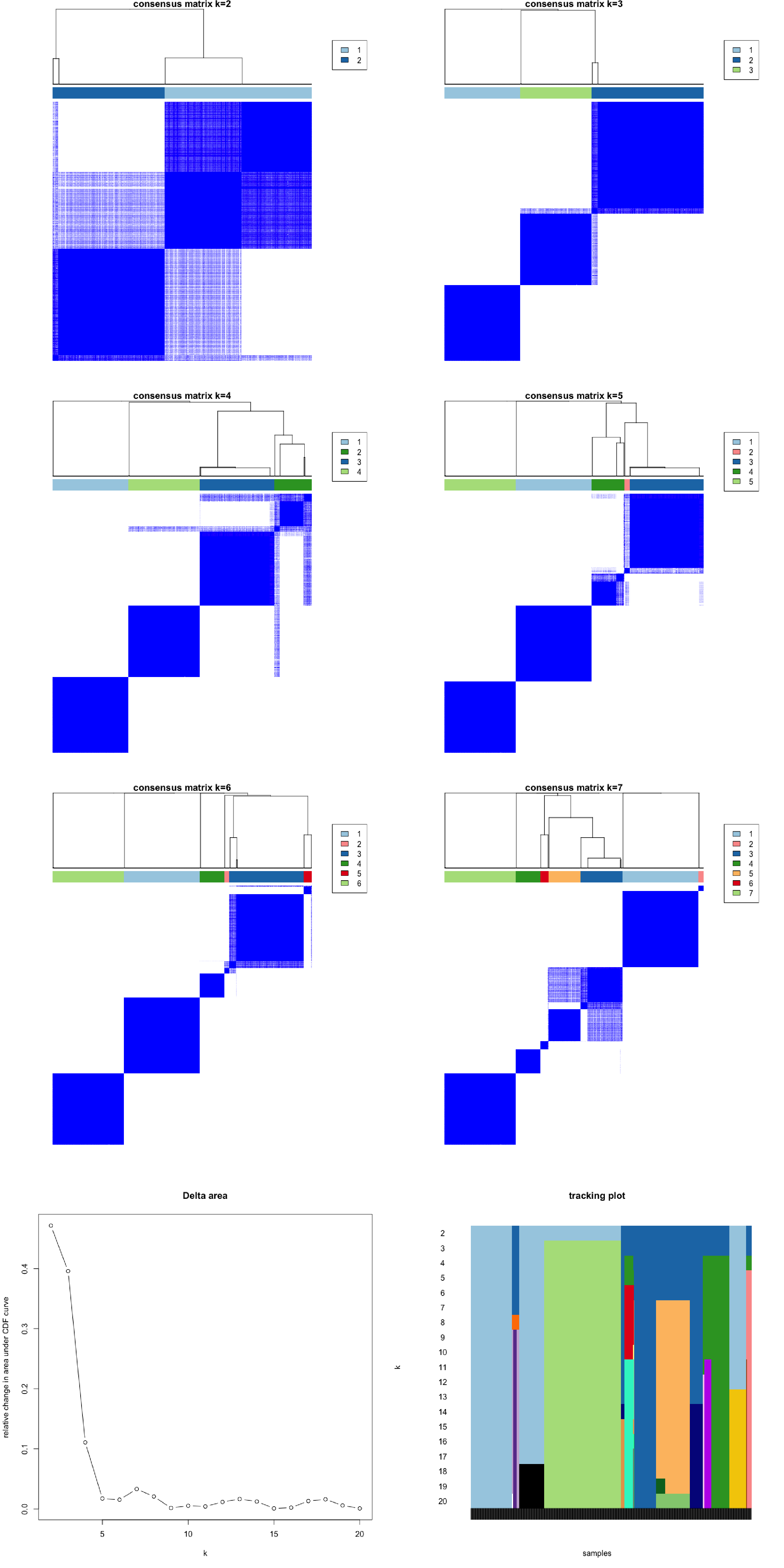

Supplement: Supplementary file 1 [file biomedicines-09-01310-s001.zip › supplementary-figures/Supp Figure S1_main_CC.png]

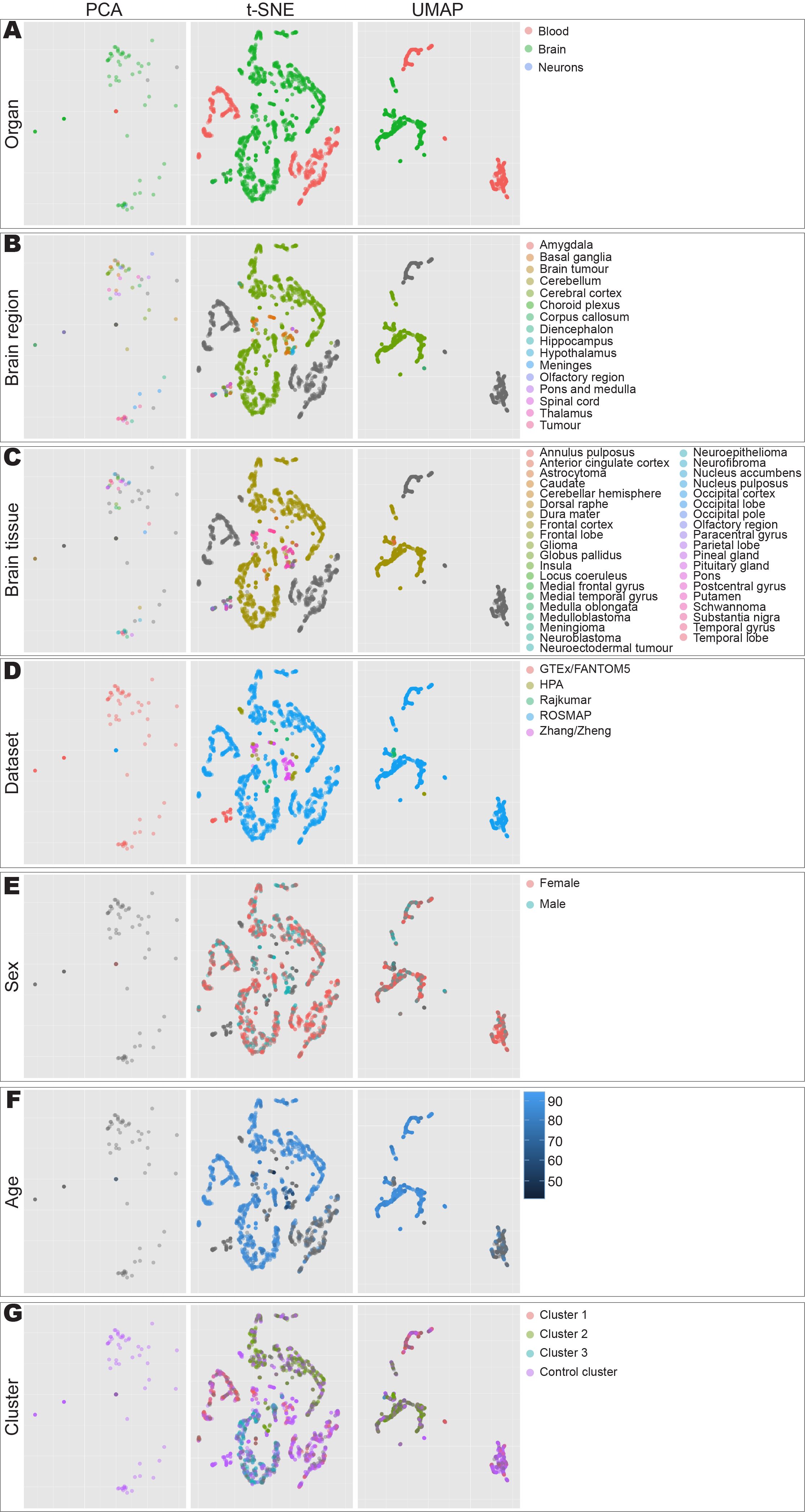

Supplement: Supplementary file 1 [file biomedicines-09-01310-s001.zip › supplementary-figures/Supp Figure S2_main_PCA-tSNE-UMAP.png]

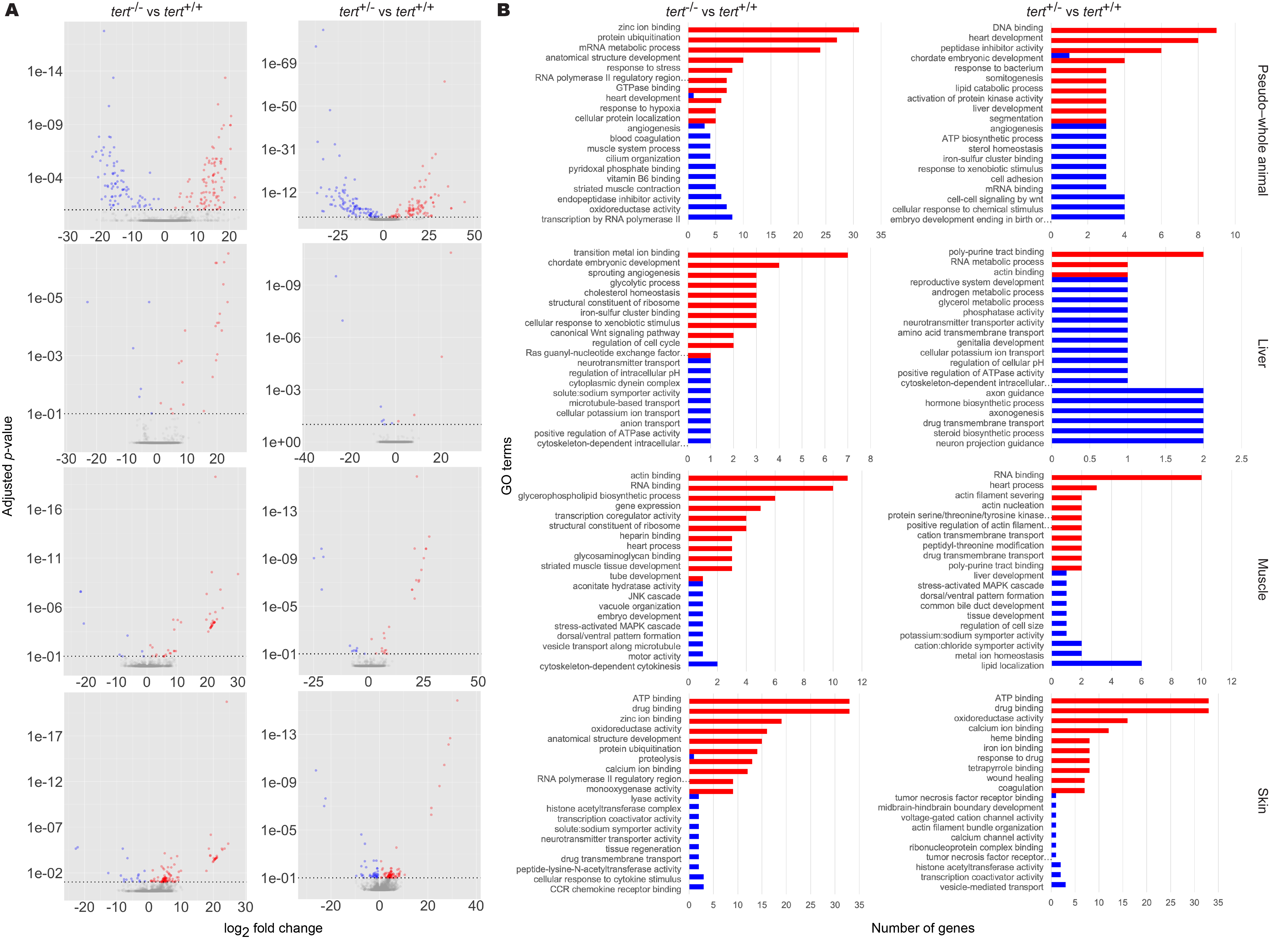

Supplement: Supplementary file 1 [file biomedicines-09-01310-s001.zip › supplementary-figures/Supp Figure S3_zeb_DEG_GSEA.png]
